# Chronic stress-induced immune dysregulation in breast cancer: Implications of psychosocial factors

**DOI:** 10.2478/jtim-2021-0050

**Published:** 2022-03-05

**Authors:** Xiuyun Chen, Mozhi Wang, Keda Yu, Shouping Xu, Pengfei Qiu, Zhidong Lyu, Xinwen Zhang, Yingying Xu

**Affiliations:** Department of Breast Surgery, the First Affiliated Hospital of China Medical University, Shenyang 110001, Liaoning Province, China; Department of Breast Surgery, Fudan University Shanghai Cancer Center, 270 Dong-An Road, Shanghai 200032, China; Department of Breast Surgery, Harbin Medical University Cancer Hospital, Harbin 150081, Heilongjiang Province, China; Breast Cancer Center, Shandong Cancer Hospital and Institute, Shandong First Medical University and Shandong Academy of Medical Science, Jinan 250117, Shandong Province, China; Breast Center, The Affiliated Hospital of Qingdao University, Qingdao 266005, Shandong Province, China; Center of Implant Dentistry, School and Hospital of Stomatology, China Medical University, Liaoning Provincial Key Laboratory of Oral Disease, Shenyang 110122, Liaoning Province, China

**Keywords:** breast cancer, psychosocial, personality, immune function, prognosis

## Abstract

Chronic stress refers to continuous emotional changes and psychological pressure that individuals experience when they are unable to adjust and stabilize the internal environment over an extended period. It can increase the pressure on endocrine mediators and cytokines in the circulation, as well as tissues throughout the hypothalamic-pituitary-adrenaline (HPA) axis and sympathetic nervous system (SNS); thus, evolving the internal environment of the tumor. This review assesses several key issues, involving psychosocial factors, and integrates clinical, cellular, and molecular studies—as well as the latest research progress—to provide a mechanistic understanding regarding breast oncopsychology. We propose that chronic stress contributes to large individual diferences in the prognosis of breast cancer survivors because they change the basic physiological processes of the endocrine and immune systems, which in turn regulate tumor growth. The study of psychological and physiological reactions of breast cancer patients suggests a new idea for psychological intervention and clinical treatment for breast cancer patients.

## Introduction

The idea that breast cancer may be associated with chronic stress dates back to approximately 200 AD when Galen pointed out that it is easier for depressed women to develop cancer than other women.^[1, 2]^ Many reviews have been published in this field, some connecting psychosocial factors to the occurrence and development of the disease, and others focused on statistics without discussing corresponding mechanism.^[3, 4]^ As cancer is a disease with biological diversity, no single psychosocial factor could be associated with all cancer development in the same way.^[5, 6, 7]^ This review combines the immune mechanism and clinical relevance of these pathways in breast cancer.

## Neuroendocrine system and breast cancer

Stressful life events, including physical stressors and psychological stressors related to the social support and ability to cope with or habituate to repeated stress, are also determined by the way a person perceives and evaluates a situation.^[8]^ In contrast to acute stress, chronic stress refers to continuous emotional changes and psychological pressure that individuals experience when they are unable to adjust and stabilize the internal environment over an extended period.^[3]^

Over the past three decades, a psychophysiological mechanism has been believed to link psychosocial factors with breast cancer through the endocrine and immune systems.^[9]^ Increasingly more evidence has shown that moderate stress can impair the immune system, promoting cancer growth.^[10]^ The nervous system receives a variety of stimuli in vivo and in vitro. It converts the stimulation into nerve impulses for conduction, thereby regulating the physiological activities of organs—including endocrine glands—and transforming appropriate responses.^[11]^ Cognitive responses from the cerebral cortex and limbic systems regulate the activities of hypothalamic and brain-stem structures, which directly control the activities of the hypothalamic-pituitary-adrenaline (HPA) axis and sympathetic nervous system (SNS).^[12, 13, 14]^

### Hypothalamic-pituitary-adrenergic axis

The HPA axis is the critical system active in stress response. The paraventricular nucleus of the hypothalamus is excited by various internal and external stimuli. Corticotropinreleasing hormone (CRH) is synthesized and then secreted into the pituitary gland to produce adreno-cortico-tropic hormone (ACTH). ACTH circulates to the adrenal cortex and releases glucocorticoids, which are involved in almost every organ system.^[15]^ Glucocorticoids can also directly or indirectly affect the growth of tumors by participating in the basic biological processes of metabolism, immune function, angiogenesis, circadian rhythm, and neuron function.

The combination of glucocorticoids and glucocorticoid receptors can stimulate the expression of antiapoptotic gene in epithelial cells and antagonize the apoptosis induced by anticancer drugs.^[16]^ Glucocorticoids promote the proliferation and survival of tumor cells.^[17, 18]^ Both chronic stress and exogenous glucocorticoid supplementation can lead to drug resistance to paclitaxel and lead to larger tumors.^[19]^ Similarly, in the culture of triple negative breast cancer cells with positive glucocorticoid receptors, the use of glucocorticoid receptor antagonists can improve the efficacy of paclitaxel-induced cytotoxicity and apoptosis.^[20, 21]^

### Sympathetic nervous system

When sympathetic nerves are excited, secretions from the adrenal medulla increase. Norepinephrine and epinephrine are released into the blood from sympathetic postganglionic fibers and the adrenal medulla. NE binds to adrenergic receptors to regulate tumor growth, progression, and metastasis.^[22, 23, 24]^ β-Adrenergic signaling may affect the development of breast cancer through several direct and indirect mechanisms, including tumor cell invasion, angiogenesis, tumor cell survival, and tumor immune interaction.^[25]^ A variety of tumors increase vascular endothelial growth factor, matrix metalloproteinase-2, and hypoxia inducible factor-1α through the cAMP protein kinase signaling pathway activated by β-adrenergic receptor, which promotes tumor angiogenesis.^[26]^ Chronic stress also increases the expression of proinflammatory cytokines IL-6 and IL-8.^[27, 28]^ In animal experiments, we found that stress-induced neuroendocrine activation had little effect on the growth of primary breast cancer, but the metastasis of distant tissues increased 30-fold.^[29]^

Chronic stress reconstituted lymphatic network plays an important role in tumor interior and surrounding, providing a pathway for tumor cell escape.^[30, 31]^ Drug inhibition of sympathetic nervous system blocks the effect of chronic stress on lymphatic remodeling in vivo and reduces lymph node metastasis in preclinical cancer models and breast cancer patients. This may be a new way of how activating the sympathetic nervous system can stimulate cancer outcomes.^[32]^

## Psychoneuroimmunology and breast cancer

**Figure 1:**
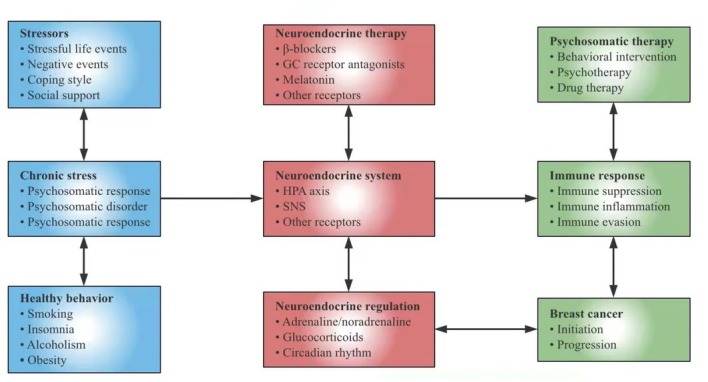
Chronic stress affects the pathogenesis of breast cancer through neuroendocrine pathways. In this model, psychosocial factors and healthy behavior regulate adrenaline, noradrenocortin, and glucocorticoid through the neuroendocrine system. Stress-responsive neuroendocrine mediators can also affect the immune system and control cell proliferation, invasion, angiogenesis, metastasis, and immune escape. In addition to explaining the biological behavioral risk factors of breast cancer, the model also proposes new targets for behavioral intervention, psychological intervention, and drug therapy. HPA: hypothalamic-pituitary-adrenaline; SNS: sympathetic nervous system; GC: glucocorticoid.

Psychoneuroimmunology refers to the mutual communication between the brain and the immune system under equal conditions, emphasizing that psychological and neural phenomena can affect the immune system. It studies how neuralgia transforms psychological factors into chronic stress that can affect healthy behavior, especially how the brain and behavior affect immune system and, in turn, are controlled by the immune system.^[33]^ The nervous system acts on the neuroendocrine system through a wide range of peripheral synapses, neurotransmitters, endocrine hormones, and cytokines secreted by nerve cells. These neurotransmitters affect immune function and their receptors act on immune cells like lymphocytes and macrophages.^[34]^ Neuroendocrine system and immune system share the same signal media and receptors, which indicates that the brain has immune regulation function and immune system has a sensory function.^[35]^ It is known that the nervous system regulates the immune system mainly through HPA and SNS (Figure 1). At present, steroid hormone receptors, catecholamine receptors, opioid receptors, peptide receptors, and other receptors regulated by neuroendocrine regulation have been found in organs and cells of the immune system. Chronic stress changes the quantity and rhythm of hormone secretion and acts on the endocrine system, most of which are immunosuppressive.^[36, 37, 38]^

Glucocorticoid is known as an immunosuppressant, which regulates the release, synthesis, and function of cytokines as well as inhibits other substances regulating immunity and inflammation. At the same time, the circadian rhythm of cortisol is also closely related to the occurrence and development of breast cancer.^[39, 40]^ Studies have shown that the diurnal rhythm of cortisol in patients with metastatic breast cancer is usually flattened or abnormal which inhibits NK cell count and NK function, resulting in early mortality.^[41]^ Thus, we speculate that NK cells may be mediators or markers of the rapid disease progression in breast cancer.

Stimulation of β-adrenergic receptors usually inhibits lymphocyte response, cytotoxicity of NK cells, and antigen extraction function of dendritic cells, and promotes tumor cell metastasis. Studies have shown that pressure activated neuroendocrine system and increased NE secretion can promote the infiltration and differentiation of CD11b+F4/80+ cells into M2 macrophages, promoting the metastasis of breast cancer cells to lymph nodes and lung while not affecting the growth of primary tumor.^[42]^

## The influence of psychological factors of breast cancer patients on tumor

Stressful life events, including physical stressors and psychological stressors related to the social support and ability to cope with or habituate to repeated stress, are also determined by the way a person perceives and evaluates a situation.^[43]^

### Stressful life events

Adverse life events include stressful life events, death of spouse, death of relatives or friends, personal health problems, divorce, income changes, and environmental changes. Many epidemiological studies have been done on whether adverse life events promote the occurrence of breast cancer. For example, because of changes in spousal relationships like divorce or death of a spouse, the risk of breast cancer after a broken marriage significantly triples.^[44]^ The study found that extreme pressure, along with low social support, was associated with a great increase in the incidence rate of breast cancer.^[45]^ However, some of the meta-analysis showed that there was no correlation between the two.^[46]^ Additionally, important interactions between behavioral stress factors and healthy behaviors, such as smoking, insomnia, alcoholism, and obesity, would further influence cancer risk.^[12, 47]^ The difference in everyone’s life experience, personality, and cognitive evaluation of life events affects the accuracy of stress assessment; therefore, it is impossible to find a clear answer from the epidemiology. However, laboratory molecular research can make up for this defect.^[48]^ The central nervous system and the limbic system of the brain become excessively tense under chronic mental stress.^[49]^ The thymus degenerates through steroid action, which not only weakens the immune function but also easily causes gene program error to manifest. This increases the sensitivity of carcinogenic factors.^[50, 51, 52]^ Studies have shown that in the face of negative life events for regional breast cancer after surgical treatment, stress levels significantly reduced NK cell lysis, NK cell response to recombinant interferon gamma (IFN-γ), and peripheral blood lymphocyte proliferation response to phytoagglutinin and monoclonal antibodies against T-cell receptors. Other work in this field has established a link between psychosocial factors and lymphocyte proliferation in breast cancer patients.^[53]^

### Emotional expression

Current researches have shown that mental illness is likely to increase the risk of death in patients with breast cancer. Depression is one of the most common mental diseases in breast cancer. A large population-based study in South Korea has found that breast cancer patients with depression and anxiety have the highest risk of death, followed by patients with depression and anxiety alone.^[54]^ The link between mental health problems and increased mortality is consistent with previous findings on breast cancer patients in an analysis of Danish population-based health registration data.^[55]^ Similarly, in another study, depression had a predicted all-cause mortality rather than breast cancer-specific mortality.^[56]^ Negative emotions may interfere with the compliance of health promotion behavior, affect the quality of life and the doctor-patient relationship, and increase the burden of physical symptoms of cancer patients.

Depression is also associated with a poor cellular immune response to specific antigens in patients with breast cancer. The relationship among depressive symptoms, cortisol secretion, and cellular immune response in 72 patients with metastatic breast cancer was investigated. Cell-mediated immunity of specific antigens was determined after intradermal administration of seven common antigens. Women with depressive symptoms showed immunosuppressive effects, such as mean induration size, while those with higher mean cortisol levels responded less to antigens.^[57]^ Previous clinical studies have shown that higher levels of cancer-specific anxiety were associated with lower NK cytotoxicity in breast cancer patients.^[58]^ In general, excessive anxiety and depression can cause the decline of immune function, reduce the activity of NK cells in the body, reduce the level of antibodies secreted by cells, and increase the chance of recurrence and metastasis of breast cancer.

### Coping style

For a long time, it has been speculated that personality traits are related to breast cancer, but study results are often inconsistent. Data from cancer patients show that those who tend to be pessimistic and negative in the face of the huge pain caused by the diagnosis and treatment of cancer may experience accelerated disease development.^[59]^ On the contrary, positive psychosocial factors, such as social support and optimism, predict longer survival.^[60]^ Coping plays an important regulatory role between stressors and stress response. Coping style mainly includes the assessment and regulation of stress and the physical or emotional response related to the event. Personality and coping style are related to a number of peripheral blood immune cells and cellular immune function. The escape coping mechanism of a breast cancer diagnosis is related to interrupted circadian rhythm, which affects the survival rate of breast cancer patients.^[61]^ For example, pain and avoidance coping are associated with interruptions to the circadian rhythm, which in turn is associated with a flattening of the daily cortisol rhythm curve in breast cancer patients before surgery. Some studies tested the coping methods and attitudes of breast cancer patients by MAC questionnaire on the day before surgery while concurrently detecting the number of lymphocytes and their subsets. It was found that negative coping styles negatively correlated with the total number of lymphocytes, B lymphocytes, CD3, and CD4.^[62]^ In metastatic breast cancer, we found that spiritual expression was associated with an increase in the number of white blood cells and circulating T cells, including Th cells and cytotoxic T cells.^[63]^ Increased NK cell activity was found in cancer patients who used humor as a coping mechanism.^[64]^

### Social support

Studies have examined the impact of social support on the survival of breast cancer patients. Compared with the social integration cohort, women diagnosed with breast cancer who experienced social isolation had a 66% increased risk of all-cause death and a three-fold increase in breast cancer-related mortality.^[65]^ A similar study, which followed young women subsequent to breast cancer diagnosis, found that larger social network correlated with lower all-cause mortality rather than breast cancer-specific mortality. However, this study focused on the number of social relationships. The quality of intimacy was found to be closely related to mortality in breast cancer patients.^[66]^ For example, when social networks are small, women with low social support have an increased risk of breast cancer-related death compared to women with high social support.

Social support can be perceived and translated into processes that alter the cellular chemistry of breast cancer.^[67]^ Social isolation can increase the risk for occurrence of and death from diseases. A large amount of evidence shows that social support may have survival value in animal and human groups. Genome-wide expression analysis in adults with increased loneliness showed upregulated expression of proinflammatory transcripts related to nuclear factor kappa B activation and downregulated expression of antiinflammatory gene expression related to glucocorticoid receptor activation.^[12]^ Additionally, the sensitivity of glucocorticoid receptor will be negatively affected, leading to a decrease in the antiinflammatory effect of glucocorticoids.^[68]^ There is a close relationship between social support and immune function. Perceived social support is conducive to increased NK activity.^[69]^

## Clinical opportunities and challenges

The comprehension of psychosocial factors on the biological and clinical significance of cancer is extending. Although the molecular pathways have not been fully described, observations to date recommend the need for new therapeutic paradigms that integrate biological behavior perspectives.

Positive psychosocial factors, such as social support, have been associated with increased cytotoxic levels of NK cells in breast cancer. With the support of society, the cytotoxicity of NK cells is not limited to the surrounding areas; it is also found in tumor-infiltrating lymphocytes, reflecting the possible psychosocial impact on the tumor microenvironment.^[70]^ Stress management interventions that inhibit chronic stress-related physiological changes may contribute to the recovery of the immune system, thereby increasing immune monitoring during active cancer treatment.^[71]^ For example, in patients with breast cancer, cognitive-behavioral stress management (CBSM) and mindfulness-based stress reduction (MBSR) can reverse the anxiety-related upregulation of circulating leukocyte proinflammatory gene expression.^[53, 72, 73]^

Similar to most medical interventions for cancer, the effectiveness of psychosocial interventions may vary with the type and stage of cancer, patient characteristics, and the type and extent of health behavior interventions. More importantly, epidemiological evidence related to psychological and social factors provides evidence for studying the biological signaling pathways and mechanisms underlying these observations. A drug intervention can be used to improve stress-related effects on cancer development and progression.

Drugs that can reestablish circadian regulation, such as melatonin, may have antitumor effects.^[74]^ In a retrospective study of patients with breast cancer, there demonstrated a 57% reduced risk of metastasis development and a 71% reduction in the risk of breast cancer-specific mortality after 10 years after adjusting for tumor size, stage, and grade in women taking any β-blocker compared to those who were not.^[24]^ The use of antidepressants may be promising, as it has been associated with certain types of cancer, along with inhibition of inflammatory response.^[75]^ Whether these agents can be used to reduce cancer risk through mechanisms related to biological behavior remains to be determined, but these studies suggest that further investigation is needed.^[76, 77]^ However, previous studies on the use of antidepressants in breast cancer have shown that there is an association between antidepressants and the risk of death is a complex issue that should be considered, such as the type of antidepressants and cancer treatment patients are receiving.^[78]^

## Discussion

Psychosocial stress can damage the immune system through neuroendocrine inhibition, leading to the growth of malignant tumors and affecting their course and outcome. The role and mechanism of chronic stress in the development of breast cancer need a model, which recognizes the relationship between the neuroendocrine and immune systems. However, the biological factors that link chronic stress with breast cancer prognosis still need to be found and verified.^[79, 80]^ At the theoretical level, considering the unusual complexity of these intermediary markers and systems, it may be necessary to test and model their relationships in a hierarchical model.^[81]^ Future studies should examine the effect of these biomarkers on the prognosis of breast cancer patients along with their related psychosocial factors and stress.^[82, 83]^

At the same time, we should not only explore the biological significance of mediators but also their clinical significance for patient health. However, chronic stress has various forms, different severity, and duration, which are difficult to control. Large-scale and well-designed studies on chronic stress related to breast cancer development are needed. Prospective studies are ideal and need to be designed with sufficient sample sizes. Multiple psychosocial factors can be examined simultaneously with objective assessments, focusing on their quality rather than cumulative effects. The impact of psychosocial factors on cancer progression has been shown to be more convincing than the onset of cancer; however, the possible cause of the expected relationship has not been identified. Although evidence is lacking regarding the main role of psychosocial factors in breast cancer development, the interactions between several psychological factors—as well as the interaction between psychological and biomedical risk factors—have rarely been studied.

Previous reports have shown that a large proportion of cancer patients with comorbidities do not receive treatment.^[84]^ It is necessary to improve the reception and recovery of mental health assessment and treatment. However, the results of psychiatric interventions that enhance effective coping and reduce affective distress seem to have beneficial effects on breast cancer survival but are not proposed as an alternative or independent treatment.^[85]^ As cancer treatment evolves toward a more patient-specific approach, we can make best of psychological factors and psychotherapy to prevent the occurrence and development of cancer as well as improve the immune status of breast cancer patients through molecular biology, so as to prolong life and improve the quality of life.
